# Changes in Sleep Quality, Depressive Symptoms, and Sleep–Wake Preference Among University Students Participating in an Eight-Week Evening Aerobic Exercise Program: A Quasi-Experimental Study

**DOI:** 10.3390/jcm15103664

**Published:** 2026-05-10

**Authors:** Şenay Demir Yazıcı, Ecem Ersungur, Nazan Öztürk

**Affiliations:** 1 Department of Physical Medicine and Rehabilitation, Faculty of Medicine, Aydın Adnan Menderes University, Aydın 09100, Türkiye; senay.demir.yazici@adu.edu.tr; 2 Söke Vocational School of Health Services, Aydın Adnan Menderes University, Aydın 09100, Türkiye; nazan.ozturk@adu.edu.tr

**Keywords:** aerobic exercise, sleep quality, depressive symptoms, university students, physical activity, morningness–eveningness

## Abstract

**Background/Objectives:** Sleep disorders and depressive symptoms are common among college students and can negatively impact quality of life. Physical activity is recommended as a non-pharmacological approach to support sleep and mental health; however, findings regarding regular evening aerobic exercise in young adults are limited. This study aimed to examine changes in sleep quality, depressive symptoms and sleep–wake preferences among university students participating in an eight-week evening aerobic exercise program. **Methods:** This quasi-experimental study included 51 university students who were assessed before and after an eight-week intervention. Participants engaged in 60 min of aerobic exercise three days a week in the evening (between 6:00 PM and 10:00 PM). Sleep quality was assessed using the Pittsburgh Sleep Quality Index (PSQI), depressive symptoms using the Beck Depression Inventory (BDI), and morning–evening type using the Morning–Evening Questionnaire (MEQ). **Results:** At the end of the eight-week period, a significant improvement was observed in total PSQI scores; significant changes were also noted in the subscales of sleep onset latency, habitual sleep efficiency, sleep disturbances, and daytime dysfunction (*p* < 0.05). A significant decrease was observed in BDI scores (median: from 17 to 9; *p* < 0.001), and the proportion of students with moderate-to-severe depressive symptoms decreased. A significant increase was also observed in MEQ scores (*p* < 0.001), which was found to be consistent with a shift toward an earlier sleep–wake preference. **Conclusions:** While these findings suggest that an eight-week evening aerobic exercise program may offer potential benefits in improving sleep quality and reducing depressive symptoms among university students, it is thought that changes in sleep–wake preferences may reflect short-term behavioral adaptation.

## 1. Introduction

Exercise, as a subset of physical activity, is a fundamental component of physical fitness and health maintenance. Unlike other energy-consuming activities, exercise involves well-planned, structured components of frequency, intensity, and duration [1]. In addition to its benefits such as increasing muscle strength and preventing injuries, exercise also has positive effects on weight control, mental health, sleep regulation, and overall quality of life [2]. To maximize the effects of exercise, biological factors must be taken into account alongside exercise itself. In this context, the concept of the circadian rhythm constitutes an important area of research focusing on biological clocks and their relationship with exercise.

Circadian rhythm refers to the approximately 24 h cyclical fluctuations in molecular, cellular, and biological processes [3]. Chronotype, which represents an individual’s preferred timing of sleep, wakefulness, and daily physical or mental activity within this rhythm, reflects interindividual differences in circadian phase [3,4]. According to chronotype, people are classified into three types: morning type, evening type, and intermediate type. Morning-type individuals prefer to wake up early and go to bed early, are more active in the early hours of the day, and perform better during these hours [5].Evening-type individuals go to bed late and wake up late. Their performance tends to be better in the afternoon or evening hours. Intermediate types are individuals who possess characteristics of both morning and evening types and are generally more active in the middle of the day. Studies show that while 40% of people are morning or evening types, approximately 60% are more inclined toward an intermediate type [4]. Chronotype is influenced by genetic, environmental, age-related, and social factors. During childhood, people tend to show more morning-type characteristics, whereas during adulthood, they become more evening-oriented, and in old age, they transition back toward morningness. Environmental factors such as geographical location, climate conditions, working hours, and caffeine consumption also affect chronotype [6]. In addition to internal biological mechanisms, chronotype and the sleep–wake cycle are also influenced by behavioral factors such as regular daily routines, physical activity programs, and social interactions. These external factors may contribute to a mismatch between the rest–activity circadian rhythm and the sleep–wake cycle and potentially lead to circadian desynchronization [7]. However, while chronotype is a relatively fixed trait, it can be “masked” by external factors such as light exposure, social obligations, or exercise; in such cases, the underlying chronotype may remain unchanged, while temporary shifts in sleep–wake timing may occur [8]. Recent studies have shown that chronotype has a significant effect on individuals’ exercise levels [8,9,10]. For example, it has been found that evening-type individuals spend a larger portion of their day sitting [9]. Similarly, studies on soccer players have shown that performance tends to peak at different times of day depending on chronotype. Therefore, exercise programs may be optimized by aligning training sessions with individuals’ inherent circadian preferences rather than attempting to modify chronotype itself [8].

The effect of circadian rhythm on exercise levels encompasses not only physical aspects but also mental and emotional aspects. Numerous studies have also shown that regular exercise has various social and psychological benefits [11,12,13]. It is known that exercise is related to mood and psychological characteristics, and that regular exercise can improve these characteristics [13]. Sleep quality is the primary indicator of overall sleep health and is usually manifested by difficulties such as trouble falling asleep, difficulty staying asleep, or frequent awakenings. The consequences of poor sleep quality are not limited to a decline in cognitive functions such as attention and memory; they also have negative effects on physical health, social relationships, and psychological well-being [14,15]. Participation in regular exercise has been suggested as one potential strategy to improve sleep quality. Although evidence shows an effect of exercise on sleep, the direction and intensity of these effects vary from person to person [1]. A meta-analysis has reported that many studies show a positive effect of physical activity on sleep. The study found that different effects may occur depending on age, gender, initial physical activity level, time period during which exercise is performed, and type of exercise [16]. However, Yang and colleagues reported moderately strong positive effects of exercise on sleep across different age groups [17]. However, the effect of these variables is not yet fully clear [18]. Therefore, demonstrating the effect of the timing and type of exercise in different populations would contribute to the literature. 

University students stand out as a group that is more vulnerable to psychological problems due to their exam preparations, irregular lifestyles, and economic situations, and this period is considered a time when they experience intense anxiety [19,20]. In addition to depression, sleep disorders are also common problems in this age group and have negative effects on both individual health and academic achievement [11,12]. Symptoms such as depression, anxiety, and stress observed in university students can lead to numerous problems ranging from academic failure to dropping out of school. Therefore, protective and preventive programs targeting these students are of great importance [21].

Exercise has long been recommended as an effective intervention tool that improves both physical and mental health [11,12,13]. Recently, it has been reported that not only the frequency and type of exercise but also the time of day it is performed has different psychological and physiological effects on individuals [13]. This situation indicates that individuals’ chronotypes may be decisive in their responses to exercise and in the effects of exercise [22]. However, studies examining the effect of exercise timing (especially evening exercise) on sleep quality and psychological symptoms in relation to sleep–wake timing remain limited in the literature.

Therefore, the aim of this study was to assess changes over time in sleep quality, depression levels, and sleep–wake preferences among university students who participated in a regular aerobic exercise program conducted in the evenings (6:00 PM–10:00 PM) over an eight-week period.

## 2. Materials and Methods

For this study, ethical committee approval was obtained from the Non-Interventional Clinical Research Ethics Committee of the Faculty of Medicine, Aydın Adnan Menderes University, Aydın, Türkiye (Approval No. 2025/98; 10 April 2025). The study was conducted in accordance with the Declaration of Helsinki. Written informed consent was obtained from all students participating in the study prior to its commencement. The authors confirm that the research complied with the ethical standards of Türkiye. The study was registered at ClinicalTrials.gov (ID: NCT06981702). The study was carried out entirely on a voluntary basis, and no incentives were provided to encourage participation.

### 2.1. Study Design

This quasi-experimental study, employing a single-group pre-test–post-test design without a control group, was conducted between April 2025 and September 2025 at the Soke School of Health Services at Adnan Menderes University in Aydın, Türkiye. Assessments were conducted one week before the start of the intervention and one week after its completion.

### 2.2. Participants

The target population of this study consisted of students enrolled in the Biomechanics course at the Soke School of Health Services, Adnan Menderes University, in Aydın. Participants were selected from among students enrolled in a single Body Mechanics course offered at a single vocational school.

All students enrolled in the course were informed about the study, and eligible students who voluntarily agreed to participate were included in the assessment. The study included students enrolled in the Body Mechanics course who voluntarily agreed to participate and had no known musculoskeletal disorders, physical disabilities, psychiatric diagnoses, or history of neurological or cardiovascular diseases. A total of 51 students were included in the study.

### 2.3. Intervention

At the beginning of the study, the researcher—a physical therapist—informed the participants about the content of the exercises, the frequency of sessions, and the fact that those who did not attend two consecutive sessions would be excluded from the study. In the study, students were asked to perform 60 min of aerobic exercise three evenings a week between 6:00 PM and 10:00 PM, including warm-up and cool-down periods. The exercise sessions consisted of a 10 min warm-up, a 40 min moderate-paced walk, and a 10 min cool-down and stretching routine [23].

The exercises were demonstrated individually by the researcher/physical therapist before the study began. A weekly schedule was created to ensure that students exercised outdoors on the same day and at the same time each week. All exercises performed by the students over the 8-week period were monitored by the researcher/physical therapist through weekly individual and group sessions. This monitoring process may have increased adherence to the program; however, it should be noted that factors such as researcher supervision and group interaction could influence participant behavior and outcomes independently of the specific effects of the exercise. Following the 8-week aerobic exercise program, students were asked to complete the MEQ, PSQI, and BDI scales again.

### 2.4. Data Collection Tools

In the study, face-to-face interviews were conducted with students to gather their demographic information. The Morningness–Eveningness Questionnaire (MEQ) was used to assess the morningness–eveningness preferences of university students before exercise, the Pittsburgh Sleep Quality Index (PSQI) was used to determine sleep quality, and the Beck Depression Inventory (BDI) was used to measure depressive symptom levels.

Demographic Information Form: Prepared by the researchers with literature support, this form includes questions about students’ sociodemographic characteristics such as age, height, weight, educational status, additional illnesses, medications they use, whether they have previously experienced sleep problems, and whether they have received any psychiatric diagnosis [24].

Morningness–Eveningness (MEQ): This is the most commonly used self-report instrument for assessing an individual’s preferred sleep–wake timing and circadian orientation. It provides an indirect estimate of chronotype based on daily activity and rest patterns. An individual’s circadian type can be understood based on their sleep and wakefulness patterns. It consists of 19 questions. The answers given to the questions on the scale are tallied and interpreted as follows: 16–41 points indicate an “evening type,” 42–58 points indicate an “intermediate type,” and 59–86 points indicate a “morning type” [25]. The Turkish validity and reliability study was conducted by Pündük et al. [26].

Pittsburgh Sleep Quality Index (PSQI): The PSQI is a self-report questionnaire that assesses overall sleep quality and seven components of sleep. These 7 components are: subjective sleep quality, sleep onset latency, sleep duration, habitual sleep efficiency, sleep disorders, sleep medication use, and daytime dysfunction. It consists of 19 items, most of which use Likert-type response formats. Using the formulas suggested by the authors, they are converted into scores for 7 components. These components include subjective sleep quality, sleep onset latency, sleep duration, and habitual sleep efficiency; the ratio of time spent in bed to time actually asleep (calculated as a percentage), sleep disorders, sleep medication use, and daytime dysfunction; and daytime impairment such as inattention and fatigue due to insomnia. Each component is scored between 0 and 3, where 0 indicates no sleep problem and 3 indicates a severe sleep problem. The PSQI total score is obtained by adding the scores of these 7 components. It ranges from 0 to 21 and indicates overall sleep quality. A total score above 5 indicates poor sleep quality [27]. The PSQI has been used in numerous studies to assess sleep quality in adult populations and examine its relationship with various outcomes [28,29]. In academic populations, the PSQI has been widely used, particularly in studies involving students [28,30]. A literature review examining factors affecting sleep quality in university students found that physical activity and healthy social relationships improve sleep quality, while caffeine consumption, stress, and irregular sleep–wake patterns reduce sleep quality [30].

Beck Depression Inventory Scale (BDI): The BDI is a self-report measure used to assess the presence and severity of depressive symptoms in individuals. The scale consists of 21 items, and each item is scored between 0 and 3, with a total score ranging from 0 to 63. As the score increases, the severity of depression also increases. The cut-off score for the scale is 17, and depression levels are classified as follows based on the total score: 0–9 points indicate normal mood, 10–18 points indicate mild depression, 19–29 points indicate moderate depression, and 30–63 points indicate severe depression [26]. The validity and reliability study of the scale in Turkish were assessed by Hisli [31].

### 2.5. Statistical Analysis

The data were analyzed using the SPSS 25.0 statistical package program (IBM Corp., Armonk, NY, USA). The distribution of continuous variables was assessed using the Kolmogorov–Smirnov test. If continuous variables show a normal distribution, they are presented as mean ± standard deviation; if not, they are presented as median (25th–75th percentile). Categorical data are presented as numbers and percentages (n, %). Paired sample *t*-tests (or Wilcoxon signed-rank tests in nonparametric conditions) were used for pre- and post-exercise comparisons. McNemar and McNemar–Bowker tests were used for comparisons of categorical variables before and after exercise. Because the study had a single-group pretest–posttest design, no independent intergroup comparisons were performed. Spearman’s correlation was used to evaluate the relationship between PSQI component scores and the total PSQI score. In all statistical analyses, *p* < 0.05 was considered the threshold for significance.

## 3. Results

A total of 51 students enrolled in a single “Body Mechanics” course at a vocational school of health services participated in the study. The participants’ mean age was 21.09 ± 3.47 years, and their mean body weight was 66.44 ± 15.80 kg. The sample was distinctly female-dominated; 40 participants (78.4%) were female, and 11 participants (21.6%) were male. Only 5 students (9.8%) lived with their families, while the vast majority, 38 students (74.5%), lived in dormitories or shared accommodations with friends. Forty-one participants (80.4%) had no underlying medical conditions and were not taking any regular medication. Regarding exercise habits, 14 individuals (27.5%) did not exercise at all, while 9 students (17.6%) exercised regularly. The participants’ demographic characteristics are presented in Table 1.

Significant changes were observed in students’ sleep–wake preferences, overall sleep quality, and depression levels in assessments conducted before and after an eight-week regular aerobic exercise program. Students’ MEQ scores were found to be a median of 41 (36–47) before exercise and 50 (45–55) after exercise, and this increase was statistically significant (*p* < 0.001). When comparing MEQ classifications before and after exercise, a statistically significant change was observed in participants’ sleep–wake preference categories (*p* < 0.001). Before the exercise, 1 participant (2.0%) was classified as a morning type, 27 (52.9%) as evening types, and 23 (45.1%) as intermediate types. After exercise, this distribution changed to 7 morning types (13.7%), 9 evening types (17.6%), and 35 intermediate types (68.6%). These results indicate a shift toward earlier sleep–wake timing and a reduction in evening preference, which may reflect behavioral changes in sleep–wake patterns over the study period. However, no significant differences in age and gender were found between the pre- and post-exercise sleep–wake preference groups. The relevant findings are presented in Table 2.

When PSQI scores before and after exercise were compared, a significant change in sleep quality was determined (*p* < 0.001). According to the PSQI total score, 37 participants (72.5%) had poor sleep quality before exercise, while this number decreased to 20 participants (39.2%) after exercise. The PSQI total score showed a median of 5 (4–8) before exercise and 4 (3–6) after exercise, and this decrease was found to be statistically significant (*p* < 0.001). Significant improvement was observed in several components of the PSQI: sleep onset latency, habitual sleep efficiency, sleep disturbances, and daytime dysfunction after exercise (*p* values were 0.0001, 0.004, 0.007, and <0.001, respectively). The relevant findings are presented in Table 3.

The median BDI score decreased from a median of 17 (12–24) before exercise to 9 (4–13) after exercise, and this change was found to be statistically significant (*p* < 0.001). When comparing BDI scores before and after exercise, a significant increase was observed in low-risk levels (normal and mild depression), while a decrease was observed in high-risk levels (moderate and severe depression), and this change was statistically significant (*p* < 0.001).

All participants who were at a “normal” level of depression risk according to their BDI scores after exercise remained at that level. Among participants with a “mild” depression risk level prior to exercise, 10 (62.5%) progressed to a “normal” level, 5 (31.3%) remained at the ‘mild’ level, and only 1 participant (6.2%) progressed to a “moderate” level. Of those at the “moderate” level, 8 (42.1%) were “normal,” 7 (36.8%) were “mild,” and 4 (21.1%) remained at the same level. Among participants at risk of “severe” depression, 3 (42.9%) improved to “mild” and 1 (14.2%) to “moderate,” while 3 (42.9%) remained at this level. The relevant findings are presented in Table 4.

The mean body weight of participants was 66.44 ± 15.80 kg before the intervention, while it was 65.36 ± 15.91 kg after the intervention. The reduction in students’ body weight after the exercise program was also found to be statistically significant (*p* = 0.003).

## 4. Discussion

The present study explored the influence of an eight-week regular aerobic exercise program performed in the evening hours (18:00–22:00) on university students’ sleep–wake patterns, sleep quality, and depressive symptom levels. Our results revealed significant improvements in sleep quality and mood, accompanied by higher MEQ scores that suggest a morning-oriented tendency. However, this change is more likely to reflect behavioral adjustments in sleep–wake timing or a masking effect rather than a true alteration in chronotype. These findings underline that regular physical activity can help young adults establish more consistent behavioral rhythms and psychological stability. These findings are important because university students encounter psychological problems more frequently due to their irregular lifestyles, economic problems, and exam stress [19,20]. Additionally, academic decisions and sleep disorders due to career planning are also common problems in this age group and have a negative impact on academic achievement [11,12,32]. Therefore, exercise-based lifestyle interventions may represent an accessible non-pharmacological strategy to enhance mental well-being and overall quality of life in this population. However, when interpreting these findings, it should be noted that the participants were selected from a single institution, consisted primarily of women (78.4%), and were enrolled in a biomechanics course; thus, they represent a convenience sample of individuals who already had an interest in body awareness and physical activity. This self-selection bias may have overestimated the observed effect sizes compared to an unselected student population.

The effect of exercise on human circadian rhythms is complex and involves both physiological and behavioral mechanisms that interact with the body’s internal clock [1]. External factors such as the timing of physical activity, light exposure throughout the day, and social routines can significantly influence circadian regulation [7].

However, exercise also has the capacity to influence circadian masking processes and may induce changes in sleep–wake timing, acting as an important non-photoperiodic timing mechanism [33]. Regular exercise interacts with the brain’s central circadian regulators, sending signals that may contribute to short-term adjustments in behavioral circadian organization [34]. This feature makes exercise a non-pharmacological behavioral tool that can help prevent or alleviate circadian rhythm desynchronization [33].

In our study, a significant increase in participants’ MEQ scores was observed following exercise, and participants exhibited a shift in their sleep–wake rhythms toward the morning. Given that the evening exercise program may have temporarily affected participants’ activity–rest cycles, this pattern is thought to represent a short-term behavioral adjustment in sleep–wake timing rather than a true modification of chronotype. Morning-type individuals have a consistent sleep–wake cycle, which leads to both better academic performance and a higher quality of life [32]. Polanska et al. reported that morning-type individuals experience a high level of well-being, while evening-type individuals have the longest sitting time during the day and are the most sedentary group. They indicated that chronotype preference and habitual activity levels are closely related, influencing both well-being and physical activity in adults [9]. Considering these findings, the post-exercise increase in MEQ scores in our study can be interpreted as improved behavioral regularity and better alignment with daily routines, rather than as a biological transformation of chronotype. A systematic review on the relationship between chronotype and physical activity and health also yielded similar results. It indicated that morning-type individuals engage in more physical activity, while evening-type individuals sit for longer periods and exhibit lower activity levels [35]. Similarly, Allee et al. also found that as individuals shifted toward the morning type, there was a significant increase in their daily step count [10]. Taken together, these results suggest that regular physical activity may promote behavioral circadian stability and psychological well-being by improving synchronization with external cues, even without altering intrinsic chronotype characteristics. For this reason, an increase in MEQ scores should be interpreted not as evidence of a permanent chronotype shift, but merely as an indicator of short-term behavioral adaptation.

Regular exercise is an effective non-pharmacological approach to improving sleep quality [36,37]. In our research results, students’ sleep quality was assessed using the PSQI total score, and a statistically significant improvement was observed after the eight-week evening exercise program. This improvement may be attributed not only to the physiological effects of exercise but also to the structured timing of the sessions, which could have stabilized participants’ daily routines and contributed to more regular sleep–wake patterns. Additionally, significant improvement was observed after exercise in several PSQI components, including sleep onset latency, habitual sleep efficiency (calculated as the ratio of time spent in bed to time actually asleep), sleep disturbances, and daytime dysfunction. These findings suggest that evening exercise, when performed regularly and at consistent hours, can promote better sleep consolidation and reduce sleep fragmentation by reinforcing behavioral consistency. The results obtained are consistent with previous studies supporting the sleep-regulating effect of exercise. Kredlow et al.’s systematic review showed that exercise improves sleep quality, shortens sleep onset latency, and increases sleep efficiency and sleep duration [16]. Similarly, Brand and Kirov reported in their studies on young adults that regular exercise positively affected sleep parameters [14]. Kalak et al. found that the exercise group showed an increase in sleep duration and efficiency and a decrease in sleep onset latency compared to the control group that did not exercise [38]. Lang has shown that more active adolescents experience longer sleep duration, less wakefulness during sleep, and higher sleep efficiency [1,39]. Animal studies also show that long-term aerobic exercise reduces sleep fragmentation and that these effects persist after exercise has ended, producing lasting effects on the central nervous system [1]. However, our study did not find a statistically significant improvement in total sleep duration. This finding differs from meta-analysis results showing that regular and acute exercise has moderate positive effects on sleep onset latency, total sleep time, and sleep quality [17]. This difference may be attributed to the age group and characteristics of the participants and the relatively short duration of our exercise program.

In addition, a statistically significant weight loss was observed in the participants throughout the study (baseline: 66.44 ± 15.80 kg; end: 65.36 ± 15.91 kg; *p* = 0.003). Although the literature reports that weight loss may have positive effects on depressive symptoms and sleep [40,41], it remains unclear whether this relationship stems from an effect independent of exercise and lifestyle changes or is a general reflection of these changes.

For this reason, the possibility that some of the observed improvements are due to physiological changes resulting from weight loss rather than the direct effects of aerobic exercise cannot be ignored. Controlling for weight change as a potential mediating factor in future studies will contribute to a more accurate assessment of the specific effects of exercise.

In our study, the prevalence of poor sleep quality was 72.5%. In a study conducted with medical students on sleep quality, poor sleep quality was reported in 52.7% of cases (average PSQI score: 6.1) [15]. The high rate of poor sleep quality observed in our study may be due to differences in the academic level and lifestyle of the selected group. Vocational school students may have limited awareness of healthy sleep habits or may lack a regular daily routine. Furthermore, the absence of a control group in our study limits the ability to attribute the observed improvements solely to the exercise intervention, as the improvement may have resulted not only from exercise but also from the increased regularity imposed by the study protocol itself. Nevertheless, the results suggest that structured behavioral interventions, even in the absence of a control group, can yield meaningful improvements in sleep quality among young adults.

Additionally, given the high prevalence of poor sleep quality at baseline (72.5%) and the predominantly female sample (80.4%), obstructive sleep apnea syndrome (OSAS) and other sleep-related breathing disorders should be considered as potential unaccounted confounding variables. OSAS may be underdiagnosed in women compared to men. Furthermore, women may experience adverse effects on cardiometabolic health, quality of life, and sleep architecture due to OSAS [42]. Therefore, it is thought that the possibility of undiagnosed sleep-disordered breathing in some participants in our study may have influenced the magnitude of the response to the exercise intervention, as measured by baseline sleep quality assessments. In future studies, the use of validated screening tools such as the STOP-BANG in samples with a predominance of women could strengthen the internal validity of sleep-related outcomes and facilitate a more accurate assessment of the effects of exercise on sleep quality [43].

The relationship between sleep and exercise is complex and is influenced by many factors, including age, gender, physical fitness, body mass index, and habitual sleep patterns, as well as the timing, intensity, and duration of physical activity [1]. In particular, the time of day when exercise is performed can act as a behavioral cue that reinforces or disrupts circadian alignment. This relationship can be better understood by conducting studies involving participants from different populations [18,44]. Our findings support the view that evening exercise performed at regular hours may serve as a stabilizing behavioral factor for sleep–wake patterns by improving perceived sleep quality through consistent behavioral changes rather than physiological alterations alone. Indeed, a meta-analysis found that while gender does not significantly alter the benefits of regular exercise on sleep, age and habitual activity level play a key role in determining the magnitude of improvement [16]. Therefore, encouraging young individuals to engage in regular, time-structured exercise may not only enhance physical health but also help regulate behavioral circadian stability and improve overall sleep quality.

In our study, no statistically significant change was observed in sleep medication use, subjective sleep quality, or sleep duration. In contrast, Yang et al. [17] found moderate-to-significant improvements in all subscales of the PSQI after exercise, except for sleep medication use, and emphasized that this effect may be influenced by the type, timing, and intensity of exercise. The absence of change in certain subscales in our study may be related to the short duration of the intervention (8 weeks) and the relatively young and healthy sample, who were less likely to use sleep medication or present severe sleep disturbances at baseline. The literature also reports that the time it takes to fall asleep increases with age, and sleep problems increase with a decrease in quality of life [10]. In young adults, however, regular exercise contributes not only to extended sleep duration but also to improved subjective sleep perception and overall restfulness [19]. Taken together, these findings suggest that even in populations with relatively normal sleep parameters, regular exercise can enhance sleep continuity and satisfaction through behavioral regulation rather than pharmacological support.

Our study found a statistically significant improvement in participants’ depressive symptom levels after exercise. An analysis of the baseline assessment in our study revealed that only 9 participants (17.6%) showed no signs of depression, while more than half exhibited moderate-to-severe depression. This result appears to be relatively high when compared to other studies conducted in Türkiye [45]. Since the sample was selected from a single college of health sciences, it is possible that this result was influenced by academic stress and living conditions. Additionally, the high baseline level may have contributed to the regression toward the mean effect. This result supports the growing body of evidence suggesting that regular physical activity contributes to reduced depressive symptoms through multiple mechanisms, including neurochemical modulation, improved sleep quality, and enhanced behavioral regulation. Haraszti et al. reported higher depressive mood, lower quality of life, and poorer perceived health in evening-type individuals [46]. Similarly, recent studies show that depression is associated with poor sleep quality and a preference for later sleep–wake patterns rather than a permanent chronotype shift [32]. The antidepressant effects of exercise are explained by increases in brain-derived neurotrophic factor (BDNF) levels and improvements in sleep quality [47,48,49,50,51]. Regular exercise improves mental health by reducing the risk of anxiety and depression; therefore, it is recommended that individual characteristics such as sleep quality and chronotype-related behavioral tendencies be taken into account when planning exercise [52]. The existing literature also supports this relationship: A study conducted in the US found that higher PSQI scores increase the risk of depressive symptoms [53], while Brand and Kirov noted that sleep quality, mood, and academic achievement are interrelated in young individuals [14]. It is known that evening chronotype individuals are more prone to depressive symptoms, and Zhou et al. have shown that sleep quality mediates this relationship [32,54]. The rapid improvement in depressive symptoms can be partly attributed to nonspecific factors such as participation in a structured program, social interaction, and the establishment of a regular routine. The absence of a control group remains a factor that leaves it unclear as to what extent these nonspecific effects can be distinguished from the specific effects of exercise. Taken together, these findings highlight that improvements in mood after exercise may result from better sleep regulation and behavioral stabilization, supporting the view that exercise acts as a behavioral synchronizer rather than altering inherent chronotype traits.

A major strength of our study is the simultaneous assessment of both physiological (e.g., weight, sleep duration, sleep onset) and psychological (e.g., depression, sleep–wake rhythm, and behavioral chronotype tendencies) parameters in participants engaged in regular exercise. The participants were students enrolled in a body mechanics course, which we believe contributed to their regular adherence to the exercise program, as they were familiar with exercise and its effects on the body. Moreover, conducting all sessions at a consistent evening time (18:00–22:00) allowed for a controlled evaluation of how regular exercise schedules can influence sleep quality and sleep–wake patterns. In addition, the exercise program was systematically supervised by a responsible physiotherapist, which ensured continuity and compliance with the intervention.

The absence of a control group in our study is one of the main limitations. This limits our ability to directly attribute the observed changes to the exercise intervention. Some of the observed improvements may be explained by factors such as behavioral consistency resulting from program participation, increased social interaction, or other factors related to the research process, rather than physical activity itself. Therefore, the findings should be interpreted as preliminary and correlational rather than causal.

An important limitation regarding the sample selection must also be considered. All participants consist of students enrolled in the “Body Mechanics” course offered at a single school of health sciences. While this constitutes a typical convenience sample, it also carries a significant risk of self-selection bias. Students enrolled in this course are likely to have a greater interest in physical activity, body awareness, and health-related behaviors compared to the general university student population. This pre-existing interest may have increased adherence rates to the exercise program and may have exaggerated the magnitude of the observed changes in sleep quality and depressive symptoms. The sample’s significant female-dominated composition (78.4%) also limits the generalizability of the findings to male students or groups with a more balanced gender distribution.

The limited sample size and the fact that the study was conducted at a single center also limit its generalizability. The significant weight loss observed in participants (baseline: 66.44 ± 15.80 kg; end: 65.36 ± 15.91 kg; *p* = 0.003) may have had an independent effect on sleep quality and depressive symptoms. A point that should not be overlooked and requires further evaluation is the possibility that this finding may have partially mediated the observed improvements. Additionally, the extension of the study period from April to September—which coincided with significant seasonal changes in daylight duration in Türkiye—may have created effects on sleep timing and mood independent of exercise. Changes in academic workload during the academic term should also be considered an uncontrolled variable that may have influenced sleep quality and levels of depressive symptoms. Additionally, the fact that participants were not screened for obstructive sleep apnea syndrome (OSAS) or other sleep-related breathing disorders prior to the study constitutes an additional limitation. Given the high prevalence of poor sleep quality at baseline (72.5%) and the female-dominated sample (78.4%), it should be considered that undiagnosed sleep-related breathing disorders may have influenced baseline sleep quality scores and the magnitude of the response to the exercise intervention [42].

The lack of long-term follow-up prevented an assessment of the sustainability of the observed changes. The fact that all exercise sessions were conducted in the evening (6:00 PM–10:00 PM) and that different time periods were not compared limits a comprehensive evaluation of the role of exercise timing in the sleep–wake cycle.

The median baseline BDI score of 17 (12–24) is interpreted to mean that the median participant was on the borderline between mild and moderate depressive symptoms. Given that this baseline level could be considered relatively high when compared to general student samples, it is believed that some of the improvements observed post-intervention in our study were due to a regression-to-the-mean effect. Furthermore, the absence of a control group makes it impossible to determine whether the reduction in depressive symptom levels is attributable to the exercise intervention or to natural fluctuations resulting from the initially high symptom levels. For this reason, changes in depressive symptoms should be interpreted as preliminary and correlational findings.

To better understand these findings and distinguish the specific contributions of exercise from other behavioral factors, larger, multicenter, randomized controlled trials are needed. Future studies should use unselected and more heterogeneous student samples, and the gender distribution should be more balanced.

## 5. Conclusions

These findings suggest that an eight-week evening aerobic exercise program may offer potential benefits. In this single-group pre-test–post-test study without a control group, changes in sleep quality, depressive symptoms, and sleep–wake preferences were observed among the students who participated in the program; the increase in MEQ scores was found to be consistent with a shift toward earlier hours. However, it is believed that this increase in MEQ scores reflects a short-term behavioral adjustment rather than a permanent chronotype shift, and this finding should be interpreted with caution. Furthermore, due to the absence of a control group, causal inferences regarding the observed changes are limited. Therefore, the findings should be interpreted with consideration of the study’s sample characteristics and design limitations. In this context, regular physical activity may represent a promising non-pharmacological approach to supporting sleep and psychological well-being in the sample of this study. Larger, more heterogeneous, multicenter randomized controlled trials are needed to confirm these findings; distinguish the specific effects of aerobic exercise from non-specific factors such as structured routines, social interaction, and researcher interest; and better understand the role of exercise type, duration, and timing in sleep–wake patterns and mental health.

## Figures and Tables

**Table 1:** Characteristics of participants.

Variable	
Age, years, mean ± SD	21.09 ± 3.477
Gender *n (%)* female male	40 (78.4%) 11 (21.6%)
Weight mean ± SD	66.44 ± 15.804
Place of residence, *n (%)* With family Dormitory With friends in a student house Alone	5 (9.8%) 38 (74.5%) 6 (11.8%) 2 (3.9%)
Existing illness diagnosed by a physician, *n (%)* No Arrhythmia Diabetes mellitus Polycystic ovary syndrome Hashimoto’s thyroiditis Scoliosis Eczema	41 (80.4%) 3 (5.9%) 3 (5.9%) 1 (2%) 1 (2%) 1 (2%) 1 (2%)
Regular medication use, *n (%)* Yes No	10 (19.6%) 41 (80.4%)
Exercise status, *n (%)* Never Occasionally Regularly	14 (27.5%) 28 (54.9%) 9 (17.6%)
SD: Standard deviation	

**Table 2:** Comparison of morningness–eveningness classification and MEQ scores before and after exercise.

	Before Exercise	After Exercise	*p*-Value
Morningness–eveningness classification *n (%)* Morning type Evening type Intermediate type	1 (2%) 27 (52.9%) 23 (45.1%)	7 (13.7%) 9 (17.6%) 35 (68.6%)	<0.001
MEQ Score	41 (36–47)	50 (45–55)	<0.001

MEQ: Morningness–Eveningness Questionnaire. Data are presented as the median (25th–75th percentile) and as a percentage.

**Table 3:** Comparison of the Pittsburgh Sleep Quality Index (PSQI) and Its Components Before and After Exercise.

PSQI Components	Before Exercise	After Exercise	*p*-Value
Subjective sleep quality	0 (0-0)	0 (0–0)	0.461
Time to fall asleep	2 (1–2)	1 (1–2)	0.001
Sleep duration	1 (0–1)	1 (0–1)	0.317
Usual sleep efficiency	0 (0–0)	0 (0–0)	0.004
Sleep disorders	1 (1–2)	1 (1–1)	0.007
Use of sleeping pills	0 (0–0)	0 (0–0)	0.461
Daytime dysfunction	2 (1–2)	1 (0–1)	<0.001
Total PSQI score	5 (4–8)	4 (3–6)	<0.001

PSQI: Pittsburgh Sleep Quality Index. Data are presented as median (25th–75th percentile).

**Table 4:** Distribution of Depression Levels Based on BDI Scores Before and After Exercise.

	Before Exercise	After Exercise	*p*-Value
According to the BDI; Normal *n* (%) Mild depression *n* (%) Moderate depression *n* (%) Severe depression *n* (%)	9 16 19 7	27 15 5 3	<0.001
BDI Score	17 (12–24)	9 (4–13)	<0.001

BDI: Beck Depression Inventory Scale. Data are presented as median (25th–75th percentile) and percentage.

## Data Availability

The data presented in this study are available on reasonable request from the corresponding author.
